# Early posttraumatic stress symptoms and levels of distress in trauma patients treated in the resuscitation room: an exploratory study

**DOI:** 10.1186/s13049-021-00830-4

**Published:** 2021-01-28

**Authors:** Iris Reiner, Manfred E. Beutel, Philipp Winter, Pol M. Rommens, Sebastian Kuhn

**Affiliations:** 1 Department of Psychosomatic Medicine and Psychotherapy, Clinic for Psychosomatic Medicine and Psychotherapy, University Medical Center Mainz, Untere Zahlbacher Str. 8, 55131 Mainz, Germany; 2 University of Applied Sciences Darmstadt, Adelungstr. 51, 64283 Darmstadt, Germany; 3 Department of Orthopedics and Traumatology, University Medical Center, Johannes Gutenberg-University, Langenbeckstr. 1, Mainz, 55131 Germany; 4 Department for Digitale Medizin, Medical Faculty OWL, Bielefeld University, Universitätsstr. 25, 33615 Bielefeld, Germany

**Keywords:** Posttraumatic stress, Prevention, Resuscitation room, Trauma

## Abstract

**Background:**

The aim of the present study was to investigate the incidence of psychological distress and posttraumatic stress symptoms in trauma patients who have been recruited from the resuscitation room. Further, we wanted to explore risk factors for posttraumatic stress symptoms, taking different accident types into account.

**Methods:**

Our sample consisted of 45 patients who have been treated in the resuscitation room and were interviewed within the first ten days after treatment. Type of accident, third party fault, previous mental health problems and pretraumatic stress were examined. Patients were interviewed with respect to their currently felt distress regarding the accident. Posttraumatic stress symptoms were measured with the German version of the Impact of Event Scale. Injury severity was assessed by means of the Injury Severity Score.

**Results:**

Our exploratory and cross-sectional project reveals that more severe injuries were associated with higher distress. However, posttraumatic stress symptoms were predicted by high distress and being involved in a car accident, but not by injury severity.

**Conclusions:**

We identified two potential risk factors for the development of posttraumatic stress in trauma patients recruited from the resuscitation room: Being involved in a car accident and high distress.

**Trial registration:**

The project has been registered at the Study Center of Mental Disorders (SPE) at the University Medical Center Mainz (No: 92072014).

## Background

Patients admitted to the resuscitation room are seriously impaired in their vital functions, have detectable injuries after trauma or have suffered a dangerous accident mechanism. A potential threat to life, serious accidents and severe injuries are risk factors for the development of posttraumatic stress symptoms. The present research explores subjectively experienced and reported levels of distress and posttraumatic stress symptoms in patients early after treatment in the resuscitation room and tries to identify risk factors for distress and posttraumatic stress symptoms.

According to ICD-10, posttraumatic stress disorder (PTSD) is a clinical condition that arises as a psychophysiological response to an extremely stressful and traumatic event of “exceptionally threatening or catastrophic nature” [1]. Symptoms include unpleasant remembering or “reliving” the trauma in the sense of intrusive flash backs, nightmares, or suffering when exposed to trauma-associated circumstances. Therefore, patients avoid (or try to avoid) trauma-associated circumstances, that they did not evade before the traumatic event. Further, patients are either not able to recall important aspects of the period of exposure to the traumatic event or show persistent symptoms of increased psychological sensitivity and arousal such as sleeping problems, irritability or hyper-vigilance [1]. Lifetime prevalence of PTSD is between 7.8 and 10.6% in the United States of America [2, 3], with presumably lower rates in Germany [4]. However, the incidence of PTSD of road traffic accident survivors is between 18 to 25% [5, 6]. Without treatment, symptoms of posttraumatic stress as well as PTSD may persist for years in a subset of trauma-exposed survivors. Chronic PTSD is associated with poor physical health, lower quality of life, impairments of social relationships and unemployment [7, 8].

The genesis of PTSD is multifactorial, with pretraumatic (e.g. psychiatric history, reported childhood abuse, and family psychiatric history), peritraumatic (e.g. severity of trauma, personal impact and proximity) and posttraumatic (e.g. lack of social support, permanent physical or financial impairments caused by trauma) aspects contributing to its development and perpetuation [9].

Previous research on subjectively experienced distress and posttraumatic stress symptoms in trauma patients have particularly focused on general motor vehicle accidents or severely injured patients, mostly a month or longer after the accident [10, 11]. However, early psychological reactions in subgroups of motor vehicle accidents (car versus motorcycle) or posttraumatic stress following injuries that needed to be treated in the resuscitation room have been poorly researched. The treatment in the resuscitation room itself can be considered as an extremely stressful event: Apart from the physical trauma or accident the patient has suffered, he or she is exposed to unknown and potentially frightening conditions such as the urgent delivery via driving or flying ambulance, unknown noises or unfamiliar movements and lights from medical machines. Yet, to our knowledge, there has been no research conducted in order to determine if patients recruited from the resuscitation room potentially experience high levels of distress or posttraumatic stress symptoms and might represent a risk group for developing PTSD as a clinical condition. Also, little is understood which groups of resuscitation room patients might suffer the most from early onset posttraumatic stress symptoms.

The first research aim was descriptive and exploratory and examined a) the level of early psychological distress, and b) posttraumatic stress symptoms in a sample of patients after treatment in the resuscitation room. Second, we explored potential risk factors that predict posttraumatic stress symptoms in patients within 10 days after treatment in the resuscitation room.

## Methods

Admission to the resuscitation room of a trauma center is indicated by the emergency medical services or emergency physician during the preclinical treatment phase. Patients transferred to the resuscitation room are seriously impaired in their vital functions or have suffered a dangerous accident mechanism or detectible injuries. In accordance to the German polytrauma protocol, all resuscitation room admitted patients undergo a standard treatment, documentation and follow-up [12].

Regular discussions in the quality circle of the Trauma Network try to identify areas of under- or overtreatment in regard to pre- and in-hospital care (diagnostics, resuscitation room treatment and in-hospital transfer). During the quality circle of the regional Trauma Network on Jan 20th 2017 trauma-related psychological disorders were identified as a potential area of undertreatment and a more systematic approach in screening for these conditions was required. As part of mandatory quality assurance and comprehensive care of our trauma center, screening for levels of distress and posttraumatic stress symptoms was planned as an exploratory study. Further systematic activities (e.g. potential implementation of distress screening as standard care in all trauma centers) designed to bring about positive changes in the delivery of care and relief of trauma-related psychological stress were aimed as a quality improvement project.

The project was screened for possible ethics issues in accordance with Dixon [13]. The proposed exploratory study was reviewed to ensure that the risks to participants were not greater than minimal and that there were appropriate protections for participants’ privacy and confidentiality of their identifiable data. Additionally, checklists from the study were stored at the study Center of Mental Disorders (SPE) at the University Medical Center Mainz. Information about background, content and aims of the study was given to eligible and available patients in understandable language by a specifically trained medical PhD student. Participation was voluntary. Oral consent to link the interview with the somatic data from the German polytrauma protocol was obtained for each individual case by the interviewer. Patient data was treated in line with General Data Protection Regulation. Regarding the data and analyses of the study, all identifying data were removed.

### Sample

All patients beyond the age of 18 who were admitted to the resuscitation room at the emergency unit of the Department of Orthopaedics and Traumatology between April and August 2017 were considered potentially eligible and screened by our team. From those 181 potentially eligible persons, a total of 45 patients (24.9%) patients could be confirmed eligible and included in the study. Potentially eligible patients were screened within 2 to 4 days after treatment in the resuscitation room. If the patient was still treated in the Department of Orthopaedics and Traumatology but could not be reached, three follow-up contact attempts within ten days after treatment in the resuscitation room were carried out before the patient was considered to drop-out. Sample recruitment procedure and drop-out reasons with number and percentages of potentially eligible participants are presented in Table 1.

**Table 1:** Recruitment and drop-outs of potentially eligible patients

*Recruitment and drop-out reason*	Eligible patients	
	n (%)	
Discharged	71	(39.2)
Transferred to other hospital units	19	(10.5)
Intensive medical care	14	(7.7)
Unreachable^a^	14	(7.7)
Deceased	7	(3.9)
Not interested in survey	5	(2.8)
Language problems	4	(2.2)
Clouding consciousness	2	(1.1)
Enrolled patients	45	(24.9)

Note. *N* = 181 ^a:^ Patients could not be reached (were not in the room) in spite of three recruitment attempts

The final sample consisted of 45 patients, 35 male and 10 female patients aged 18 to 73 years (Mean: 43.3; SD = 17.7), who were interviewed by a medical PhD student. Age, length of hospital stay and accident type of the total sample and for subsamples of males and females are shown in Table 2.

**Table 2:** Age, accident type and length of hospital stay

Variables	Total sample ***N*** = 45		Males ***n*** = 35		Females ***n*** = 10	
*Age (years)*	*M (SD)*					
	43.3	(17.7)	44.4	(5.4)	41.3	(4.0)
*Length of hospital stay (days)*	*M (SD)*					
	10.7	(7.9)	11.9	(8.3)	6.4	(4.6)
*Accident Type*	*n (%)*					
Motorcycle accident	15	(33.3)	14	(31.1)	1	(2.2)
Falling accident	10	(22.2)	7	(15.6)	3	(6.6)
Car accident	9	(20)	6	(13.3)	3	(6.7)
Bicycle accident	7	(15.6)	5	(11.1)	2	(4.4)
Other accidents^a^	4	(8.9)	3	(6.7)	1	(2.2)

*Note.*^*a:*^ Other accident types involved mainly industrial accidents

*M* Mean, *SD* Standard Deviation

#### Materials

- The overall injury burden was assessed by means of the Injury Severity Score, abbreviated ISS [14], as the sum of the squares of the highest Abbreviated Injury Scale (AIS) code in each of the three most severely injured ISS body regions. The AIS describes all lesions in a 6-point ordinal scale ranging from minor (1) to untreatable (6) injury and distinguishes between anatomically based body regions. The ISS is dimensional and ranges from 0 to 75.

- Posttraumatic stress symptoms were assessed with the German version of the Impact of Event Scale [15–17]. Respondents were asked to focus on the index trauma, the recent accident and resuscitation room experience. The Impact of Event Scale (IES) consists of 15-items that inquire on a 0 (= not at all) to 4 (= extremely) scale symptoms of posttraumatic stress, categorized as two subscale clusters, namely intrusion and avoidance. The sum of the two subscales is the total stress score (IES- total score) mirroring the occurrence of posttraumatic stress symptoms. - Levels of distress were assessed with an analogous 1 to 10 distress scale (“how distressed do you feel regarding your accident that has been treated in the resuscitation room?”) ranging from 1 (= not distressed at all, absolutely calm and happy) to 10 (= worst distress ever experienced), adapted from the Subjective Units of Distress Scale by Wolpe [18]. The level of distress scale is a numeric scale. Concerning clinical impact, we considered a cut-off point of 5 and patients scoring 6 and above as experiencing “high distress”.

- Pretraumatic stress as a dichotomous variable (yes or no) was surveyed by means of the interview question: „Did you experience any stress life events in the past six months?” If required, the interviewer was prompted for further clarification.

- Accident characteristics were examined in both interview and from the patient’s record: Third party fault (yes or no) in the course of the accident as well as type of accident (see also Table 2). For further analyses the following dichotomous variable were used: Motorcycle accident (yes or no), car accident (yes or no), falling accident (yes or no).

- Previous mental health problems as dichotomous variable (yes or no) were investigated by means of the question: “Have you ever received a definite diagnosis of any depressive or anxiety disorder (medical history of depression or anxiety disorder)?”

### Statistical analysis

We reported absolute numbers, percentages, means with standard deviations as well as minimums and maximums. To investigate relations between groups, for example between gender and third party fault, Chi Square tests were used. ISS and IES Scales were treated as quasi-interval data. Bravis-Pearson Correlation coefficients were carried out to indicate the strength and direction of relationships between two variables. To compare groups with respect to continuous scales such ISS and IES scales, independent samples t-tests were conducted. Linear regression analyses were conducted to predict posttraumatic stress symptoms. *P*-values are based on two-tailed tests and are presented as exact values. Two-sided *p*-value of 0.05 or less was considered as statistically significant. No adjustments for multiple testing were performed, as this study was an exploratory. All statistical analyses were performed using SPSS Version 24 (IBM Corporation, Armonk, NY, USA).

## Results

### Descriptive data

Possible influences of age and gender on severity of injury, posttraumatic stress symptoms, level of distress, third party fault, accident type and previous mental health problems were tested via t-tests, chi-square tests and Pearson correlations, revealing no significant association between age, gender and any of the variables. With regards to the ISS, scores ranged in our sample from 1 (minimum) to 41 (maximum), with a mean of 10.2 and standard deviation of 8.06. Distributions of ISS scores in severity of injury groups according to Copes and colleagues [19] and all dichotomous variables that influence posttraumatic stress symptoms for total sample as well as subsamples (males and females) are presented in Table 3:

**Table 3:** Previous mental health problems, pretraumatic stress, third party fault and ISS groups

Variables	Total sample ***N*** = 45		Males ***n*** = 35		Females ***n*** = 10	
	*n (%)*					
Pretraumatic stress (yes)	14	(31.1)	10	(22.2)	4	(8.9)
Previous mental health problems (yes)	11	(24.4)	8	(17.8)	3	(6.7)
Third party fault (yes)	10	(22)	7	(15.6)	3	(6.7)
Severity of injury (ISS value intervals)						
Mild injury (1–8)	8	(17.8)	6	(13.3)	2	(4.4)
Moderate injury (9–15)	13	(28.9)	9	(20)	4	(8.9)
Major injury (16–24)	13	(28.9)	10	(22.2)	3	(6.7)
Severe injury (25 and above)	11	(24.4)	10	(22.2)	1	(2.2)

### Levels of distress and posttraumatic stress

Table 4 presents descriptive statistics of levels of distress and posttraumatic stress symptoms (IES, total score). Regarding level of distress, the full 1–10 range was exhausted. Regarding posttraumatic stress symptoms, the minimum score was 0 and the maximum score was 44. Mean and standard deviations indicate great variability of posttraumatic stress symptoms among the patients. Additional analyses showed that 16 patients (35.6%) scored 26 and higher in reference to posttraumatic stress scores reflecting a moderate or severe impact [16]. 24 patients (53.4%) scored 6 and above in reference to levels of distress and experienced therefore high distress.

**Table 4:** Levels of distress and posttraumatic stress symptoms

	*M*	*SD*	*Minimum*	*Maximum*
Distress	6.1	2.6	1	10
Posttraumatic Stress	10.36	12.83	0	44

*Note*. *N* = 45. *M* Mean, *SD* Standard Deviation

ISS scores were not significantly associated with previous mental health problems, pretraumatic stress, third party fault or accident type. In order to investigate relationships between ISS, distress and posttraumatic stress symptoms (IES-total score), Bravis-Pearson correlation coefficients were carried out. As presented in Table 5, distress but not posttraumatic stress symptoms were significantly associated with severity of injury. Distress was also significantly correlated with posttraumatic stress symptoms.

**Table 5:** Intercorrelations between severity of injury (ISS), levels of distress and posttraumatic stress symptoms (IES)

	ISS	Distress
IES	.25	.37*
ISS	–	.43**

Note. N = 45; *p *<* .05; **p < .01

### Risk factors for the development of posttraumatic stress symptoms

In order to investigate predictors for posttraumatic stress symptoms, a linear regression analysis was conducted. Results are displayed in Table 6, R^2^ = .46, *F* (8.44) = 3.85, *p* < .01.

**Table 6:** Regression model of severity of injury, high distress, pretraumatic stress, third party fault previous mental health and accident types on posttraumatic stress symptoms (IES total score)

	*B*	*SE*	*β*	*T*	*p*
Severity of injury	.29	1.78	.02	.16	.87
Distress	1.40	0.68	.29	2.04	.05*
Pretraumatic stress	.95	4.10	.03	.23	.82
Third party fault	−3.51	4.06	-,11	−.86	.39
Previous mental health problems	−3.29	4.56	−.11	−.72	.47
Motorcycle accident (yes)	6.39	4.01	.24	1.60	.12
Car accident (yes)	13.20	5.45	.35	2.42	.02*
Falling accident (yes)	−4.00	5.12	−.13	−.78	.44

Note. N = 45; *p < .05

Among all main predictors of posttraumatic stress symptoms in the regression model, significant effects were observed for distress (*β*  = .29, *t* = 2.04, *p* = .05) and being involved in a car accident (*β*  = .35, *t* = 2.42, *p* = .02).

## Discussion

First, our investigation revealed that a high rate of patients recruited from the resuscitation room suffer from severe distress and posttraumatic stress symptoms. Within ten days after treatment in the resuscitation room, 53.4% of participating patients reported serious psychological distress as measured by a distress score above 5.

Second, we identified distress and being involved in a car accident as risk factors for the development of posttraumatic stress symptoms.

Interestingly, higher severity of injury was not directly linked with higher levels of posttraumatic stress symptoms. There is empirical evidence that subjective responses to injury, e.g. emotional distress or negative appraisal of the traumatic event, rather than “objective” parameters such as the ISS account substantially for the pathogenesis of PTSD [20, 21]. Our results fit into the cognitive model of PTSD [22] which claims that negative appraisals particularly in the initial phase after trauma exposure contribute to the development of PTSD.

Findings of our investigation also underline that patients being involved in a car accident might be vulnerable for the development of PTSD. Previous studies have shown that patients involved in a motor vehicle accident are highly prone to the development of posttraumatic stress [23]. Most studies, however, pool together motor vehicle accidents, regardless of the type of vehicle. Our results show that not only the type of accident, but also the type of vehicle might influence the psychological processing of the accident. Further research involving larger samples of patients involved in subgroups of motor vehicle accidents (car vs. e.g. motorcycle accidents) needs to be conducted.

Clinical implications of our results are clear and easy to implement: Patients who have been treated in the resuscitation room need to be screened for psychological distress, independent of injury severity. This screening should be carried out after treatment in the resuscitation room and when patients are out of a life-threatening situation. They should simply be asked how distressed they feel on a 1 to 10 scale. According to our study, special attention should be also given to car accident patients as they are particularly vulnerable. Patients with a level of distress score of five and above as well as car accident patients could be offered psychosomatic counseling (if allowed by hospital conditions), psychoeducational information or further contact points. Early interventions addressing stress management (e.g. breathing relaxations), self-care and possible negative appraisals about the injury and treatment in the resuscitation room have been proven to be effective in the long-term prevention of depression and posttraumatic stress symptoms [24]. In vulnerable patients and those suffering from more severe posttraumatic stress symptoms, short-term interventions such as brief cognitive behavioral therapy may reduce subsequent mental health problems and prevent a chronic posttraumatic stress [25, 26].

Limitations of the results are noteworthy. In our study, we assessed posttraumatic stress symptoms but not PTSD per se. Consequently, we cannot speak of PTSD as a clinical diagnosis. Moreover, the clinical diagnosis may not be confirmed before four weeks after the traumatic event [1]. Our investigation is limited with regards to the small sample size. Only 25% of trauma patients who were admitted in the resuscitation room were interviewed. The high proportion of patients who had been already discharged could not be screened. This might lead to a substantial bias in our results. In order to screen discharged patients, future studies might explore distress and posttraumatic stress in a sample of patients treated in the resuscitation room who have been discharged shortly after treatment. Patients who still needed intensive care or who were transferred to other units of the hospital were also not interviewed. However, our focus was to explore early psychological reactions. Therefore, an extended recruiting time period was not possible.

Finally, prospective and longitudinal study designs are necessary to investigate how patients recovered from posttraumatic stress or developed symptoms afterwards. Although we identified two risk factors (car accident and distress), it is important to keep in mind that posttraumatic stress symptoms are likely multi-causal. Many types of traumata (e.g. non-sexual physical violence, sexual trauma, natural disasters) were not studied. Trauma patients with differing risk factors and post-trauma psychosocial and physical circumstances may come to express posttraumatic symptoms through individual-specific pathways.

## Conclusion

We conclude that high levels of distress and particularly the involvement in a car accident increase the probability to develop posttraumatic stress symptoms in resuscitation room patients. As result from our exploratory study and a quality improvement of trauma care, routine screening for levels of distress linked with an offer for psychosomatic counseling for distressed patients was initiated at all centers of our Trauma Network.

In the future, randomized controlled trial studies are needed in order to evaluate the effectiveness of screening these patients for distress and of respective psychosomatic counseling in the prevention of posttraumatic stress disorder.

## Data Availability

The datasets used and analyzed during the current study are available from the corresponding author on reasonable request.

## Abbreviations

AIS: Abbreviated Injury Scale
IES: Impact of Event Scale
ISS: Injury Severity Score
PTSD: Posttraumatic Stress Disorder
